# Eccentric contractions disrupt FKBP12 content in mouse skeletal muscle

**DOI:** 10.14814/phy2.12081

**Published:** 2014-07-17

**Authors:** Cory W. Baumann, Russell G. Rogers, Nidhi Gahlot, Christopher P. Ingalls

**Affiliations:** 1 Department of Kinesiology and Health, Muscle Biology Laboratory, Georgia State University, Atlanta, 30302, Georgia

**Keywords:** Damage, force, mouse, recovery, skeletal muscle

## Abstract

Strength deficits associated with eccentric contraction‐induced muscle injury stem, in part, from impaired voltage‐gated sarcoplasmic reticulum (SR) Ca^2+^ release. FKBP12 is a 12‐kD immunophilin known to bind to the SR Ca^2+^ release channel (ryanodine receptor, RyR1) and plays an important role in excitation‐contraction coupling. To assess the effects of eccentric contractions on FKBP12 content, we measured anterior crural muscle (tibialis anterior [TA], extensor digitorum longus [EDL], extensor hallucis longus muscles) strength and FKBP12 content in pellet and supernatant fractions after centrifugation via immunoblotting from mice before and after a single bout of either 150 eccentric or concentric contractions. There were no changes in peak isometric torque or FKBP12 content in TA muscles after concentric contractions. However, FKBP12 content was reduced in the pelleted fraction immediately after eccentric contractions, and increased in the soluble protein fraction 3 day after injury induction. FKBP12 content was correlated (*P *= 0.025; *R*^2^**= 0.38) to strength deficits immediately after injury induction. In summary, eccentric contraction‐induced muscle injury is associated with significant alterations in FKBP12 content after injury, and is correlated with changes in peak isometric torque.

## Abstract (precis)

Eccentric contraction‐induced muscle injury is associated with immediate and prolonged strength deficits that stem in part from impaired sarcoplasmic reticulum (SR) calcium release. The content of FKBP12, a 12‐kD immunophilin known to bind to the SR calcium release channel and influence SR calcium release, is reduced in mouse skeletal muscle immediately after injury induction and is significantly associated with strength deficits.

## Introduction

Unaccustomed exercise is known to injure skeletal muscle. It is generally accepted that eccentric contractions, which decelerate limbs during exercise are the primary cause of muscle injury due to their intrinsic capacity to produce high levels of stress. Eccentric contraction‐induced skeletal muscle injury is characterized by damage to sarcomeres and plasma membranes, inflammation and swelling, release of skeletal muscle proteins into circulation, reduced range of motion, and delayed onset muscle soreness and stiffness (Clarkson and Hubal 2002; Warren et al. 2002b; Allen et al. 2005; Hyldahl and Hubal 2013). Functionally, eccentric contraction‐induced muscle injury is associated with immediate and long‐lasting reductions in muscle strength (Lowe et al. 1995; Ingalls et al. 1998a,b; Warren et al. 1999; Rathbone et al. 2003). Ultimate recovery of peak muscle strength can take weeks and requires the upregulation of protein synthesis and satellite cell activity (Lowe et al. 1995; Rathbone et al. 2003). Given the magnitude and duration of the functional deficits caused by eccentric contractions, it is likely that multiple mechanisms contribute to the loss and recovery of force production.

Although impaired voltage‐gated sarcoplasmic reticulum (SR) Ca^2+^ release is known to contribute to strength deficits early (i.e., 0–3 day) after exercise‐induced muscle injury (Balnave and Allen 1995; Ingalls et al. 1998b), the exact mechanism of SR dysfunction remains unclear. In skeletal muscle, excitation‐contraction (E‐C) coupling can be defined as Ca^2+^ release from the SR after the voltage‐sensitive dihydropyridine receptor (DHPR) embedded in the T‐tubule directly activates the ryanodine receptor 1 (RyR1) located in the SR membrane. RyR1, a large homotetrameric protein complex, interacts with numerous proteins (e.g., DHPR, FKBP12, junctophilin, calmodulin, calsequestrin) that serve to regulate SR Ca^2+^ release (Chelu et al. 2004; Kushnir et al. 2010; Lanner et al. 2010). We have shown that eccentric contractions reduce junctophilin 1 and 2 (JP1 and JP2), proteins that normally maintain close apposition of T‐tubule and SR membranes, and the loss of these proteins contribute to early strength deficits (Corona et al. 2013). JP proteins may be reduced immediately after performance of eccentric contractions via Ca^2+^‐dependent proteolysis given the elevation in cytosolic [Ca^2+^] (Balnave and Allen 1995; Lynch et al. 1997; Ingalls et al. 1998b) and the potential of calpain cleavage of JP proteins (Murphy et al. 2013). Decreases in JP content are associated with increased distance between DHPR and RyR1, and decreases in voltage‐gated SR Ca^2+^ release (Ito et al. 2001; Komazaki et al. 2002; Hirata et al. 2006; Golini et al. 2011). Therefore, strength deficits associated with eccentric contraction‐induced muscle injury may stem from less efficient signaling between the DHPR and RyR1 proteins.

Alternatively, strength deficits stemming from SR dysfunction after the performance of eccentric contractions may also result from alterations in intrinsic RyR1 function. In support of this notion, drug‐induced SR Ca^2+^ release rates are significantly reduced immediately after the performance of eccentric contractions (Ingalls et al. 1998b) although RyR1 protein content is unaltered (Ingalls et al. 2004). Given the large number of proteins known to interact with RyR1, it is possible that eccentric contractions may disrupt binding of these critical proteins and alter RyR1 function. One such protein is FKBP12, a 12 kDa protein that binds the immunosuppressive drugs FK506 and rapamycin. Normally, FKBP12 binds to the subunits of the RyR1 homotetramer, and stabilizes a closed state of the SR Ca^2+^ channel and prevents channel opening to subconductance states (Brillantes et al. 1994; Ahern et al. 1997). Decreasing FKBP12 content via genetic modifications reduces voltage‐gated SR Ca^2+^ release in myotubes (Avila et al. 2003; Tang et al. 2004). Furthermore, Bellinger et al. (2008) demonstrated chronic exhaustive exercise depletes RyR1 of FKBP12, promotes leaky RyR1 channels and enhances fatigability, and drugs that prevent the removal of FKBP12 attenuated these detrimental effects. Although chronic exhaustive exercise is associated with FKBP12 depletion and SR Ca^2+^ leak (Bellinger et al. 2008), there have been no studies that have examined the effects of eccentric contractions on FKBP12 content in skeletal muscle. Therefore, the purpose of this study was to test the hypotheses that eccentric contractions disrupt FKBP12 content in mouse skeletal muscle, and that the loss of FKBP12 is associated with skeletal muscle dysfunction.

## Material and Methods

### Animals

Female C57BL6 mice 2–4 months old were used in this study. The mice were housed in groups of 5 animals per cage, supplied with food and water ad libitum, and maintained in a room at 20–22°C with a 12‐h photoperiod. Mice were euthanized with an overdose of isoflurane followed by either cervical dislocation or thoracotomy. All animal care and use procedures were approved by the institutional animal care and use committee and met the guidelines set by the American Physiological Society.

### Experimental design

**Figure 1.**
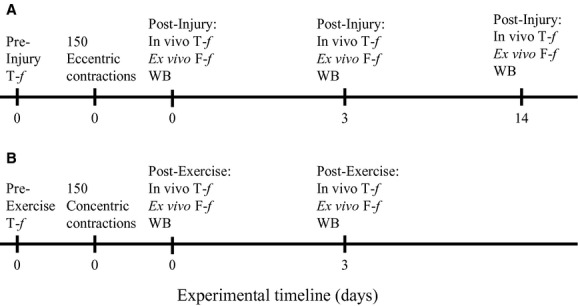
Experimental time line for the eccentric (A) and concentric (B) contraction protocols. T‐*f,* isometric torque as a function of frequency; F‐*f*, isometric force as a function of frequency. WB, western blot.

The overall experimental design of the study is illustrated in Figure 1. Mouse anterior crural muscle [tibialis anterior (TA), extensor digitorum longus (EDL), and extensor hallucis muscles] strength was assessed in vivo before and after a single bout of 150 concentric or eccentric contractions. Specifically, isometric torque produced by this muscle group as a function of stimulation frequency (20–300 Hz) was measured in anesthetized mice before and immediately after (*n *= 38) the eccentric contraction bout, and in subgroups of these mice at 3 (*n *= 13) and 14 (*n *= 10) days. Due to the rapid recovery of mouse anterior crural muscle strength following concentric contractions (Warren et al. 1999), isometric torque was measured before and immediately after (*n *= 16) the concentric contraction bout, and then only at 3 days (*n *= 8) in a subgroup of these mice. Preexercise torque served as control for all in vivo measurements.

FKBP12 protein content was determined in the left TA muscle from mice that performed either 150 concentric or eccentric contractions and in the nonexercised contralateral TA muscles, which served as controls for the Western blot analyses. Therefore, FKBP12 content was determined in the TA muscles immediately after (*n *= 7) the performance of eccentric contractions, and at 3 (*n *= 8) and 14 (*n *= 8) days. FKBP12 content was also determined in the TA muscles immediately after (*n *= 4) the performance of concentric contractions, and at 3 (*n *= 6) days. Nonexercised contralateral TA muscles (*n *= 15) served as controls. FKBP12 can be bound to either the RyR1 protein or free in the cytosol, therefore the TA muscle homogenate was centrifuged to obtain a pellet fraction containing RyR1 and a supernatant fraction containing free FKBP12. FKBP12 protein content in each fraction was assessed via Western blot.

Isometric force production of isolated EDL muscle as a function of stimulation frequency (10–300 Hz) and caffeine force was assessed ex vivo from control mice (*n *= 10) that performed no in vivo exercise, and mice that had previously performed concentric (0 [*n *= 8] or 3 day [*n *= 8] post) or eccentric (0 [*n *= 11], 3 [*n *= 8], or 14 day [*n *= 8] post) exercise. EDL muscle exposure to 2 mmol L^−1^ caffeine was used to assess potential changes in the sensitivity of RyR1 to caffeine at concentrations known to induce twitch potentiation. Because 50 mmol L^−1^ caffeine elicits SR Ca^2+^ release by acting directly on RyR1 and therefore by‐passes the upstream components of E‐C coupling (Herrmann‐Frank et al. 1999; Lamb et al. 2001), relative reductions in 50 mmol L^−1^ caffeine induced‐ and electrically stimulated‐force were compared to serve as an indirect marker of E‐C coupling failure (Warren et al. 1993; Ingalls et al. 1998b; Corona et al. 2010).

### Experimental methodology

#### In vivo muscle strength analysis and injury induction

**Figure 2.**
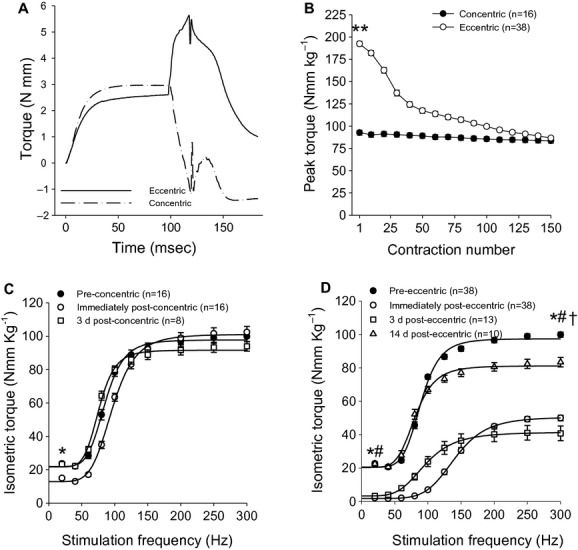
Representative torque output as a function of time during a single contraction during the eccentric and concentric contraction protocols (A; Note: the rapid spikes at the end of the movements correspond to inertia artifacts of the lever arm), peak in vivo torque produced during 150 eccentrics or concentric contractions (B), isometric torque as a function of stimulation frequency after either 150 concentric contractions (C) or eccentric contractions (D). Isometric torque measurements were made before (Pre), immediately (0 d) and 3 days (3 d) after the concentric contraction bout (C), and immediately, 3 days and 14 days (14 d) after the eccentric contraction bout (D). Isometric torque data were modeled with equation 1, listed in Material and Methods. *Torque immediately after exercise is significantly different from preexercise; ^#^torque 3 days after exercise is significantly different from preexercise; ^†^torque 14 days after exercise is significantly different from preexercise; **significantly different from concentric. Values are means ± SE.

Contractile function (i.e., torque–frequency relationship) of the left anterior crural muscles was measured in vivo immediately before and after, as well as 3 or 14 days after a single bout of 150 eccentric contractions, and 3 days after a bout of 150 concentric contractions as previously described (Lowe et al. 1995; Ingalls et al. 1998a,b; Warren et al. 1999; Corona et al. 2008a,b; Corona and Ingalls 2013). Briefly, mice were anesthetized with isoflurane (1.5% isoflurane and 400 mL O_2_ per min) and placed on a temperature controlled platform to maintain core body temperature between 35 and 37°C. The left knee was clamped and the left foot was secured to an aluminum “shoe” that is attached to the shaft of an Aurora Scientific 300B servomotor (Aurora, ON, Canada). Sterilized needles were inserted through the skin for stimulation of the left common peroneal nerve. Stimulation voltage and needle electrode placement were optimized with 5–15 isometric contractions (200 ms train of 0.1 ms pulses at 300 Hz). Contractile function of the anterior crural muscles was assessed by measuring peak isometric torque as a function of stimulation frequency (20–300 Hz). The anterior crural muscles performed either concentric or eccentric contractions through a 38° angular movement at 2000 s^−1^ starting from a 19° plantarflexed or dorsiflexed position, respectively. The eccentric and concentric contractions were preceded by a 100 ms isometric stimulation (Fig. 2A).

#### Ex vivo analysis of EDL muscle

Extensor digitorum longus muscles were dissected free and studied using an ex vivo preparation as previously described (Ingalls et al. 1998a,b, 2004; Corona et al. 2008a, 2010; Corona and Ingalls 2013). The muscles were mounted in an organ bath containing a Krebs‐Ringer bicarbonate buffer (pH 7.4) with (in mmol L^−1^) 144 Na^+^, 126.5 Cl^−^, 6 K^+^, 1 Mg^2+^, 1 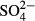, 1 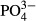, 25 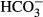, 1.25 Ca^2+^, 0.17 leucine, 0.10 isoleucine, 0.20 valine, 10 glucose, and 10 *μ*g mL^−1^ gentamicin sulfate and 0.10 U mL^−1^ insulin (the buffer was equilibrated with 95% O_2_–5% CO_2_ gas). The distal tendon was attached by silk suture and attached to a fixed support, and the proximal tendon was attached to the lever arm of a servomotor system (Aurora Scientific 300B). Optimal physiological muscle length (*L*_o_) in the chamber was set with a series of twitch contractions (0.2 ms pulse at 150 V). Peak isometric force as a function of stimulation frequency (10–300 Hz) was measured at 35°C during isometric contractions (200 ms trains of 0.2 ms pulses), with 3 min between contractions. Caffeine sensitivity and peak caffeine contracture force were assessed by measuring changes in baseline force during exposure to caffeine (2 and 50 mmol L^−1^) and twitch contractions at a rate of 0.2 Hz. Force (N) produced by the EDL muscle was normalized to physiological cross‐sectional area (PCSA) as described previously (Lowe et al. 1995; Ingalls et al. 1998a,b, 2004; Corona et al. 2008a, 2010; Corona and Ingalls 2013).

#### Western blotting

Following dissection, skeletal muscles were weighed, frozen in liquid nitrogen, and stored at −72°C until further use. Muscles were homogenized in a buffer of the following constituents (in mmol L^−1^): 250 sucrose, 100 KCl, 20 MOPS, and 5 EDTA (pH 6.8). Because FKBP12 can exist attached to RyR1 or as a soluble protein, muscle homogenate was centrifuged at 10,000 ***g*** for 15 min at 4°C to obtain a pelleted fraction and a soluble protein fraction. Protein content was determined spectrophotometrically using the Bradford assay. Pelleted (100 *μ*g) and soluble (150 *μ*g) protein fractions were loaded onto a 12% polyacrylamide gel (Acrylamide to Bis‐; 37.5:1) and separated according to molecular weight (100 V for 30 min; 150 V for 45 min). The protein was then transferred to a nitrocellulose membrane (constant 1.3 A, up to 25V for 7 min). Following transfer, the membrane was blocked overnight at ~4°C in 5% nonfat dried milk (w/v) in tris‐buffered saline with 0.1% Tween‐20 (TBS‐T). The following morning, the membranes were then probed with rabbit anti‐mouse FKBP12 primary antibody (PA1‐026; Thermo Scientific, Rockford, IL) for 2 h at room temperature on an orbital shaker. FKBP12 antibody was diluted to 1:1667, while GAPDH antibody (MAB374; Millipore, Billerica, MA) was diluted 1:1000 in TBS‐T. Following incubation in primary antibody, membranes were washed with TBS‐T (3 × 15 min) and then probed with horseradish peroxidase conjugated goat anti‐rabbit IgG secondary antibody (G21234; Invitrogen, Grand Island, NY) diluted in TBS‐T (1:20,000) for 1 h at room temperature with shaking. The membranes were then washed as described above and treated with enhanced chemilumescent solution (Thermo Scientific) prior to detection using a BioRad ChemiDoc imaging station (Hercules, CA). FKBP12 optical density from the soluble protein fraction was normalized to the GAPDH content, whereas FKBP12 from the pelleted fraction was normalized to the protein band corresponding to actin from a Ponceau‐stained membrane due to the lack of GAPDH in this fraction.

#### Data modeling and statistics

Torque– and force–frequency relationships were modeled with the following equation: 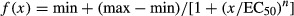


Where *x* is the stimulation frequency, min and max are the smallest (i.e., twitch) and largest (i.e., peak tetanic) respective forces estimated. EC_50_ is the stimulation frequency at which half the amplitude of force (max – min) is reached and *n* is the coefficient describing the slope of the steep portion of the curve.

Torque– and force–frequency parameters, anthropometric measurements, and protein content values were analyzed separately using a one‐way ANOVA. Eccentric and concentric contractions during each type of exercise bout, as well as pre‐ and postexercise protocol force values were analyzed using a group by contraction or time split‐plot ANOVA. Post hoc means comparisons testing was performed when a significant ANOVA was observed. Statistical significance was achieved with *P* ≤ 0.05. All statistical testing was performed with SigmaPlot (San Jose, CA).

## Results

### Body weight

The body weight of the control mice (22.3 ± 0.4 g) was significantly less (*P *= 0.02) than the weight of the mice in the concentric (24.4 ± 0.6 g) and eccentric (24.3 ± 0.4 g) exercise groups. This body weight difference is explained by a difference in age; mice in the control group were approximately 6 weeks of age, whereas, the age of the other mice was approximately 8–10 weeks. The initial body weight of mice performing in vivo contraction protocols (i.e., concentric and eccentric) were not different (*P *= 0.59) from each other. Although there were no significant differences (*P* ≥ 0.076) in body weight among any of groups after concentric and eccentric contraction protocols, isometric torque produced by anterior crural muscles was normalized to body weight to allow comparisons of torque with our previous published studies.

### 150 eccentric and concentric contractions in vivo

Peak torque produced by anterior crural muscles during the first contraction of the eccentric contraction protocol was 108% greater (*P* < 0.001) than peak torque produced during the first contraction of the concentric contraction protocol (Fig. 2B). Although peak torque significantly (*P* < 0.001) decreased over the 150 contractions in both eccentric and concentric contraction protocols, the decrease in peak eccentric torque (54.6 ± 1.2%) was greater (*P* < 0.001) than the reduction in peak torque for the concentric contractions (9.5 ± 2.1%).

### In vivo isometric torque

Before performing concentric and eccentric contractions, there were no differences (*P* ≥ 0.20) in normalized isometric torque (i.e., 20–300 Hz) or Hill equation coefficients between groups of mice (Fig. 2C and D, Table 1). Performing 150 concentric contractions resulted in low‐frequency fatigue as we have shown previously (Warren et al. 1999). Peak isometric twitch torque was reduced 38.5 ± 2.6% (*P *= 0.001) immediately after concentric exercise but had returned to normal values by 3 days (*P *= 0.97). Peak isometric tetanic torque (i.e., 300 Hz) was not significantly (*P *= 0.29) reduced at any time following concentric exercise (Fig. 2C). The stimulation frequency that produces 50% of peak isometric tetanic torque (EC_50_) was increased (*P *= 0.02) immediately after the concentric contractions, and returned to preexercise values by 3 days (*P *= 0.93) (Table 1).

**Table 1.** Anterior crural muscle in vivo isometric torque parameters

	Concentric			Eccentric			
	Pre	0 day	3 day	Pre	0 day	3 day	14 day
Sample size	16	16	8	38	38	13	10
Body weight (g)	24.4 ± 0.6	24.4 ± 0.6	23.9 ± 0.5	24.3 ± 0.4	24.3 ± 0.4	23.7 ± 0.5	23.9 ± 0.7
Min_meas_ (N mm kg^−1^)	23.7 ± 1.0	14.4 ± 0.7†	23.3 ± 1.0	22.6 ± 0.6	2.3 ± 0.2*	4.4 ± 0.8*	22.0 ± 1.0
Max_meas_ (N mm kg^−1^)	99.9 ± 3.2	102.6 ± 3.4	93.9 ± 2.9	98.8 ± 1.5	50.2 ± 1.4*‡	43.6 ± 3.9*‡	84.9 ± 2.5**
Min_estim_ (N mm kg^−1^)	21.8 ± 0.9	12.2 ± 0.5†	21.7 ± 1.0	20.6 ± 0.6	1.9 ± 0.2*	4.2 ± 0.8*	19.7 ± 0.8
Max_estim_ (N mm kg^−1^)	97.5 ± 3.3	101.0 ± 3.6	91.5 ± 3.0	96.5 ± 1.4	50.8 ± 1.4*‡	44.3 ± 3.8*‡	82.7 ± 2.4**
EC_50_ (Hz)	85.0 ± 1.7	97.1 ± 1.5†	76.0 ± 1.7	88.2 ± 0.9	140.1 ± 1.5‡	94.5 ± 2.4	82.0 ± 2.3
*n* coefficient	6.53 ± 0.2	5.83 ± 0.2	6.65 ± 0.2	6.62 ± 0.2	6.38 ± 0.2	4.54 ± 0.2γ	5.82 ± 0.3

Values are means ± SE. The minimum (Min) and maximum (Max) torques measured and estimated represent twitch and peak tetanic torques, respectively. EC_50_ is the stimulation frequency at which half of the rise in amplitude of torque occurred. The *n* coefficient describes the slope of the steep portion of the torque–frequency curves depicted in Figure 2. Pre, 0 day, 3 day, and 14 day: time course of contractions, that is, before and immediately, 3 or 14 days after, respectively.

† Significantly different from Concentric Pre and 3 day.

* Significantly different from Eccentric Pre and 14 days.

‡ Significantly different from Eccentric Pre, 0 and 3 days.

** Significantly different from all other Eccentric groups.

γ Significantly different from Eccentric Pre and 0 day groups. *P* ≤ 0.05.

Performing 150 eccentric contractions injured the anterior crural muscles as evidenced by long‐lasting maximal strength deficits. Peak isometric twitch torque was reduced immediately (92.1 ± 0.8%; *P* < 0.001) and 3 (86.3 ± 4.1%; *P* < 0.001) days after eccentric exercise, and peak isometric tetanic torque (i.e., 300 Hz) was reduced immediately (51.6 ± 1.3%; *P* < 0.001), 3 (60.7 ± 3.9; *P* < 0.001), and 14 (16.1 ± 2.6%; *P *= 0.003) days after exercise (Fig. 2D, Table 1). The isometric twitch and tetanic strength deficits were greater (*P* < 0.001) after performance of eccentric than concentric contractions. The slope coefficient (*n*) of the isometric torque–stimulation frequency relationship was significantly (*P* < 0.001) less than preinjury by 3 days and returned to normal by 14 days (*P *= 0.24; Fig. 2D, Table 1). EC_50_ was greater (*P* < 0.001) than preinjury immediately after the eccentric and concentric contraction protocols, and had returned to normal values by 3 (*P* ≥ 0.08) days after both exercise protocols and remained unchanged (*P *= 0.22) 14 days posteccentric contractions (Table 1).

### Ex vivo EDL muscle force after 150 eccentric or concentric contractions

**Figure 3.**
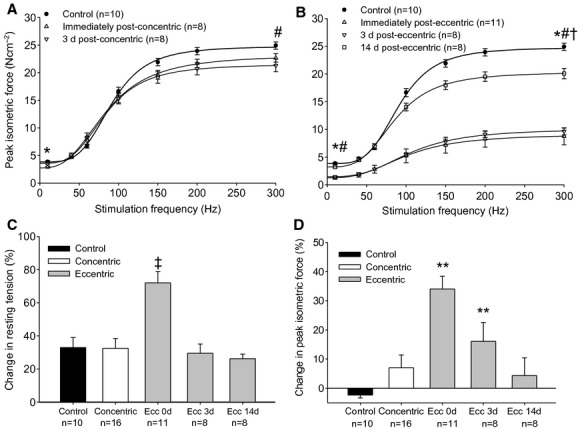
Ex vivo extensor digitorum longus (EDL) muscle isometric force as a function of stimulation frequency after the concentric (A) and eccentric (B) contraction bouts. Isometric force data were modeled with equation 1, listed in Material and Methods. Percent changes in resting tension (C) and peak submaximal isometric force (D) during 3 min of 2 mmol L^−1^ caffeine exposure. Isometric force measurements were made in control muscles and in muscle immediately (0 d) and 3 days (3 d) after the concentric contraction bout, and immediately, 3 days and 14 days (14 d) after the eccentric contraction bout. Concentric contraction groups (0 and 3 d) were combined in C and D. *Force immediately after exercise is significantly different from control; ^#^force 3 days after exercise is significantly different from control; ^†^force 14 days after exercise is significantly different from control; ^‡^significantly different from all other values; **significantly different from control. Values are means ± SE.

Similar to anterior crural muscle function, EDL muscle experienced modest low‐frequency fatigue following 150 concentric contractions. Peak isometric twitch force (*P*_t_) and the slope coefficient of the force–stimulation frequency relationship were less (*P* ≤ 0.007) than uninjured control muscle immediately after concentric exercise, whereas peak isometric tetanic force (*P*_o_), EC_50_, change in resting tension, and peak caffeine contracture force were not different (*P* ≥ 0.17) than controls at this time (Fig. 3A, Table 2). By 3 days after concentric exercise, minor contractile dysfunction was still evident by decreases (*P* ≤ 0.02) in *P*_o_ (15%) and the slope coefficient compared to control muscles.

**Table 2.** Extensor digitorum longus (EDL) muscle ex vivo isometric force parameters

	Control	Concentric		Eccentric		
		0 day	3 day	0 day	3 day	14 day
Sample size	10	8	8	11	8	8
EDL (mg)	9.6 ± 0.3	11.9 ± 0.2*	11.1 ± 0.3*	11.8 ± 0.2*	12.0 ± 0.3*	10.7 ± 0.2
*L*_o_ (cm)	1.40 ± 0.01	1.44 ± 0.02	1.41 ± 0.01	1.38 ± 0.02	1.41 ± 0.03	1.42 ± 0.02
Min_meas_ (N cm^−2^)	3.86 ± 0.2	2.89 ± 0.2*	3.55 ± 0.2	0.87 ± 0.1**	1.88 ± 0.1**	3.21 ± 0.2
Max_meas_ (N cm^−2^)	24.9 ± 0.7	22.7 ± 0.8	21.3 ± 1.1*	7.0 ± 0.6**	9.7 ± 0.5**	20.1 ± 0.9*
Min_estim_ (N cm^−2^)	3.85 ± 0.2	2.8 ± 0.1*	3.6 ± 0.2	0.9 ± 0.1*	1.4 ± 0.1*	3.2 ± 0.2
Max_estim_ (N cm^−2^)	24.8 ± 0.6	23.1 ± 0.7	21.5 ± 1.1*	7.5 ± 0.7**	10.2 ± 0.5**	20.3 ± 0.9*
EC_50_ (Hz)	91.4 ± 1.7	85.3 ± 3.6	83.6 ± 3.5	116.1 ± 5.3**	109.0 ± 4.8**	86.1 ± 1.1
*n* coefficient	4.2 ± 0.1	2.9 ± 0.04*	3.5 ± 0.1*	3.0 ± 0.1*	3.0 ± 0.1*	3.5 ± 0.1*
50 mmol L^−1^ Caffeine (N cm^−2^)	6.5 ± 0.25	6.1 ± 0.14	6.3 ± 0.24	4.7 ± 0.15*	4.8 ± 0.12*	6.5 ± 0.2
50 mmol L^−1^ Caf/*P*_o_	27.3 ± 0.73	26.4 ± 0.51	30.0 ± 1.20	70.0 ± 4.35†	50.6 ± 1.93‡	32.6 ± 1.03

Values are means ± SE. *L*_o_ is optimal muscle length coinciding with peak twitch force; Minimum and maximum forces measured (meas) and estimated (estim) represent twitch and peak tetanic forces, respectively. EC_50_ is the stimulation frequency at which half of the rise in amplitude of force occurred. The *n* coefficient describes the slope of the steep portion of the force‐frequency curves depicted in Figure 3. EDL, extensor digitorum longus muscle. Pre, 0 day, 3 day, and 14 day: time course of contractions, that is, before and immediately, 3 or 14 days after, respectively.

* Significantly different from Control.

** Significantly different from Control, Concentric 0 and 3 days, and Eccentric 14 days.

† Significantly different from Control, Concentric 0 and 3 days, Eccentric 3 and 14 days.

‡ Significantly different from Control, Concentric 0 and 3 days, Eccentric 0 and 14 days. *P* ≤ 0.05.

The performance of eccentric contractions caused significant injury to the EDL muscle. *P*_t_ and *P*_o_ were 79% and 72% less (*P* < 0.001) than uninjured control EDL muscles immediately after exercise, respectively, and these strength deficits of 62–65% persisted (*P* < 0.001) through 3 days (Fig. 3B, Table 2). Moreover, the strength deficits caused by eccentric contractions were greater (*P* < 0.001) than those produced by concentric contractions. Muscle function improved by 14 days after injury induction, but *P*_t_ and *P*_o_ were still less (*P* ≤ 0.03) than uninjured control values. Compared with uninjured muscle, the slope coefficient of the force–stimulation frequency relationship was significantly (*P* < 0.001) reduced immediately, 3 and 14 days after eccentric exercise. EC_50_ was greater (*P* ≤ 0.03) in EDL muscles that performed eccentric contractions than control and concentric exercise immediately and 3 days after injury.

Subjecting uninjured EDL muscles to 2 mmol L^−1^ caffeine causes minor increases (*P *= 0.03) in resting tension but does not result in significant (*P *= 0.72) potentiation of the 40 Hz contractile response (Fig. 3C and D). The increase in resting tension (72 ± 6.8%) observed immediately after eccentric contractions was significantly (*P* ≤ 0.003) greater than all other conditions (26–35%). Although peak submaximal isometric force was not augmented (*P* ≥ 0.29) by 2 mmol L^−1^ caffeine in EDL muscles from control and concentric contraction conditions, this submaximal force increased (*P* ≤ 0.04) immediately (34.1%) and 3 days (16.1%) after performing eccentric contractions. Peak 50 mmol L^−1^ caffeine contracture force was significantly (*P* < 0.001) lower immediately and 3 days after eccentric contractions compared with caffeine force from control and concentric muscles (Table 2). When comparing peak caffeine contracture and isometric tetanic force deficits to estimate E‐C coupling failure (Ingalls et al. 1998b; Corona et al. 2010), approximately 44% and 49% of the isometric force deficits observed immediately and 3 days injury induction, respectively is explained by EC uncoupling.

### Muscle wet weights and protein contents

Swelling of the anterior crural muscles is a characteristic of exercise‐induced muscle injury and is reflected by changes in wet weight of muscle in this study. When compared to its contralateral control muscle, the injured TA muscle exhibited greater (*P* ≤ 0.007) wet weight at 0 and 3 days after performance of eccentric contractions, whereas control muscles exhibit no difference (*P *= 0.22) in their weights (data not shown). In general, the left EDL and TA muscle wet weights of the control mice were significantly (*P* ≤ 0.04) less than the weights of nearly all other groups except for muscles that were allowed to recover 14 days (*P* ≥ 0.62) after eccentric contractions (Tables 2 and 3). There were no significant differences (*P* ≥ 0.55) in absolute (mg per muscle) or relative (% of wet weight) total protein content of TA muscles among the control, concentric, or eccentric groups (Table 3). Compared with control TA muscle, there was a significant (*P *= 0.02) decrease in relative pellet protein content 3 days after eccentric contractions (Table 3). Absolute soluble protein content of TA muscles was significantly reduced (*P* ≤ 0.02) 14 days after eccentric contractions compared to all other groups except for control (*P *= 0.12). In contrast, relative soluble protein content of TA muscles was only significantly (*P* ≤ 0.03) reduced 14 days after eccentric contractions compared to concentric contractions but not the other eccentric contraction (*P* ≥ 0.15) or control (*P *= 0.06) groups (Table 3).

**Table 3.** Tibialis anterior (TA) muscle wet weight and protein parameters

	Control	Concentric		Eccentric		
		0 day	3 day	0 day	3 day	14 day
Sample size	10–22	4–8	4–8	8–15	8–11	8–10
TA muscle weight (mg)	40.9 ± 0.9	40.9 ± 0.8	43.4 ± 0.6*	44.2 ± 0.6*	45.4 ± 0.5*	40.9 ± 1.3
TA/body weight (mg g^−1^)	1.8 ± 0.03	1.7 ± 0.06	1.8 ± 0.04	1.8 ± 0.04	1.9 ± 0.04δ	1.7 ± 0.04
TA total protein (mg per muscle)	14.6 ± 0.6	13.6 ± 0.9	14.9 ± 1.0	15.2 ± 0.5	15.2 ± 0.7	13.4 ± 0.6
TA total protein (% wet wt.)	35.3 ± 1.3	32.9 ± 2.2	34.3 ± 2.4	35.2 ± 1.1	33.3 ± 1.4	32.0 ± 1.2
TA pellet protein (mg per muscle)	6.0 ± 0.3	5.6 ± 0.7	6.6 ± 0.8	5.8 ± 0.3	4.8 ± 0.5	5.4 ± 0.7
TA pellet protein (% wet wt.)	15.2 ± 0.8	13.9 ± 2.2	15.1 ± 1.9	13.3 ± 0.9	10.5 ± 1.2*	12.9 ± 1.1
TA soluble protein (mg per muscle)	5.0 ± 0.1	5.3 ± 0.1	5.5 ± 0.1	5.3 ± 0.2	5.3 ± 0.2	4.4 ± 0.3†
TA soluble protein (% wet wt.)	12.1 ± 0.2	12.8 ± 0.3	12.6 ± 0.4	12.1 ± 0.4	11.7 ± 0.5	10.6 ± 0.6‡

Values are means ± SE. Tibialis anterior muscle protein content was determined in whole muscle homogenate (total) and supernatant (soluble) and pellet fractions after centrifugation. Pre, 0 day, 3 day, and 14 day: time course of contractions, that is, before and immediately, 3 or 14 days after, respectively. Control includes nonexercised contralateral TA muscles.

* Significantly different from Control.

δ Significantly different from Concentric 0 day and Eccentric 14 day.

† Significantly different from all other groups except Control.

‡ Significantly different from all other groups except Eccentric 3 day (*P* ≤ 0.05).

### FKBP12 content

**Figure 4.**
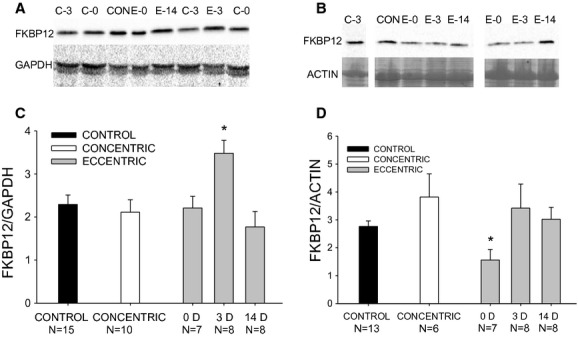
FKBP12 protein content in the supernatant (A and C) and pellet (B and D) of TA muscle after in vivo concentric (“C”) and eccentric (“E”) contractions. FKBP12 was normalized to GAPDH in the supernatant (A and C) and to the protein band corresponding to actin in the pellet (B and D). FKBP12 was determined via Western blot in unexercised control muscle, and in muscles immediately (0 d), 3 days (3 d) and 14 days (14 d) after either concentric or eccentric contractions. FKBP12 content was not different between the two concentric groups, and the data were combined. *Significantly different from Control. Values are means ± SE. TA, Tibialis anterior.

We estimate that approximately 60% of the FKBP12 was found in the pellet fraction, which is in agreement with observations that approximately 60–75% of FKBP12 is normally found in the pellet fraction after centrifugation in mouse skeletal muscle (Susan Hamilton, unpublished observation). FKBP12 content in the pellet fraction normalized to actin protein was not significantly different between control and concentric muscles (*P *= 0.11). However, the performance of eccentric contractions resulted in a 43% decrease (*P *= 0.02) in normalized FKBP12 content in the pellet fraction immediately after the eccentric contractions (Figs. 3D, 4B). Normalized FKBP12 content in the pellet had returned to control values by 3 and 14 days (*P* ≥ 0.51; Figs. 3D, 4B).

FKBP12 content in the supernatant fraction normalized to GAPDH protein was not significantly different between control and concentric muscles (*P *= 0.61). Although eccentric contractions did not affect normalized FKBP12 content immediately after exercise, it was significantly (*P *= 0.03) elevated 52% by 3 days postinjury and then had returned to control values by 14 days (*P *= 0.65; Fig 4A and C).

FKBP12 content in the pellet fraction immediately after injury induction was correlated (*P *= 0.025) with deficits in maximal isometric torque (*R*^2^ = 0.38). However, there were no significant (*P* ≥ 0.32) correlations between FKBP12 content and isometric strength when analyzed over all the time points.

## Discussion

Unaccustomed exercise that requires significant use of eccentric contractions is known to induce focal damage to force‐generating (i.e., sarcomeres) and transmitting (i.e., cytoskeleton) structures. Increasing evidence indicates that eccentric contractions can also damage triad and E‐C coupling structures (Warren et al. 1993, 1995, 1999, 2002b; Balnave and Allen 1995; Takekura et al. 2001; Yeung et al. 2002; Corona et al. 2010). Given the various loci of cellular damage and long duration of functional impairment, strength deficits associated with eccentric contraction‐induced muscle injury most certainly stem from multiple mechanisms. However, it appears that damage to E‐C coupling components is immediate and pervasive within skeletal muscle following injurious exercise, accounting for significant deficits in skeletal muscle force production (Warren et al. 1993; Balnave and Allen 1995; Ingalls et al. 1998b; Corona et al. 2010). The primary findings of this study indicate that FKBP12 content within skeletal muscles of mice is disrupted by the performance of eccentric contractions. Given the role of FKBP12 in regulating RyR1 function, it is not surprising that immediate reductions in FKBP12 content are correlated with decreases in skeletal muscle function.

In this study, regression analysis indicates that 38% of the variability in isometric tetanic torque immediately after the eccentric contraction bout can be accounted for by changes in FKBP12 in the pellet fraction. We have shown that eccentric contractions reduce both voltage‐gated SR Ca^2+^ release and drug‐induced SR Ca^2+^ release rates immediately after exercise (Ingalls et al. 1998b). Moreover, decreases in FKBP12 content has been shown to impair voltage‐gated SR Ca^2+^ release in myotubes (Avila et al. 2003; Tang et al. 2004). A loss of FKBP12 from RyR1 has also been associated with strength deficits associated with muscle fatigue resulting from chronic exhaustive exercise (Bellinger et al. 2008). In contrast to these findings, we previously demonstrated that muscle‐specific knockdown of FKBP12 from birth resulted in less isometric tetanic force deficits immediately after eccentric contractions in EDL muscles from adult mice (Corona et al. 2008b). It is possible that adaptions in the EDL muscle to the long‐term reduction in FKBP12 contributed to the resistance to eccentric contraction‐induced muscle injury. In support of this idea, muscle‐specific knockdown of FKBP12 is associated with an upregulation of the slow myofiber phenotype (Tang et al. 2004), which is less susceptible to eccentric contraction‐induced muscle injury (Warren et al. 1994).

The decrease in FKBP12 content in the pellet fraction immediately after the performance of eccentric contractions also occurred at the same time that resting tension in the EDL muscle was disrupted. The 72% increase in resting tension during the 2 mmol L^−1^ caffeine exposure likely results from increases in resting cytosolic [Ca^2+^], which may stem from leaky RyR1 channels. Bellinger et al. (2008) reported that the exhaustive exercise eliminated FKBP12 from RyR1 resulting in leaky SR Ca^2+^ channels and reduced SR Ca^2+^ stores. We have shown that resting free cytosolic Ca^2+^ concentration is increased immediately after eccentric contraction‐induced muscle injury (Ingalls et al. 1998b). Elevated cytosolic Ca^2+^ may have contributed to enhanced submaximal contractile potentiation observed in the EDL muscle during the 2 mmol L^−1^ caffeine exposure immediately after injury induction by increasing Ca^2+^‐calmodulin mediated myosin light chain phosphorylation. To this end, Rijkelijkhuizen et al. (2005) reported that the magnitude of submaximal contractile potentiation was approximately doubled compared to uninjured rat skeletal muscle after the performance of eccentric contractions.

Alterations in FKBP12 content in the TA muscle may stem from posttranslational modification (e.g., phosphorylation, S‐nitrosylation) or enzymatic cleavage of RyR1, which removes FKBP12 from the RyR1 subunit. Once removed from RyR1, FKBP12 may be lost from the myofiber similar to other muscle proteins (e.g., CK, LDH, and MHC) presumably via disruptions in the integrity of T‐tubule and plasma membranes (Warren et al. 1995; Takekura et al. 2001; Yeung et al. 2002). RyR1 is a known substrate for Ca^2+^‐dependent proteolysis (Gilchrist et al. 1992; Wu et al. 1997; Shevchenko et al. 1998). Moreover, Murphy et al. (2013) have recently shown that supraphysiological activation of skeletal muscle results in Ca^2+^‐dependent proteolysis of JP1 and 2, and impaired voltage‐gated SR Ca^2+^ release. Although eccentric contraction‐induced muscle injury is associated with elevated cytosolic Ca^2+^ and calpain activation, pharmacological manipulation of Ca^2+^ in our mouse model has not affected the magnitude of injury or force deficits (Lowe et al. 1994; Warren et al. 2002a; Ingalls et al. 2004).

Previous reports indicate that chronic fatigue in mouse skeletal muscle is associated with FKBP12 depletion from RyR1 via increased phosphorylation and/or S‐nitrosylation of RyR1 (Bellinger et al. 2008). Moreover, intense cycling exercise in humans resulted in increased levels of phosphorylation and nitrosylation of RyR1, and FKBP12 depletion of RyR1 in quadriceps muscle (Bellinger et al. 2008). A single bout of maximal eccentric contractions has also been shown to increase phosphorylation of RyR1 in human skeletal muscle for 30 min after the exercise (Gehlert et al. 2012). The extent of phosphorylation and S‐nitrosylation of the RyR1 in mouse skeletal muscle after performance of eccentric contractions is unknown. Although our previous work indicates that nitric oxide synthesis protects skeletal muscle from exacerbation of strength deficits in our mouse injury model (Corona and Ingalls 2013), it is possible that eccentric contractions promotes phosphorylation and/or S‐nitrosylation of RyR1 and may alter FKBP12‐RyR1 binding.

Three days after injury induction, FKBP12 content in the supernatant was significantly increased and content in the pellet fraction returned to normal values (Fig. 4). Although this change in FKBP12 content did not affect voltage‐gated force output in injured skeletal muscle at this time, the increase in resting tension observed during exposure to 2 mmol L^−1^ caffeine was significantly reduced. Moreover, it is possible that the return of FKBP12 in segments of the myofiber that are not undergoing phagocytosis at 3–5 days may facilitate the recovery in E‐C uncoupling and explain paradoxical recovery of peak isometric force at a time when myofibrillar protein is being lost (Ingalls et al. 1998a). Bellinger et al. (2008) reported that drug treatment designed to restore FKBP12 binding to RyR1 was effective at preventing exercise‐induced increases in RyR1 open probability and minimizing strength loss associated with fatigue. The increase in FKBP12 at 3 days postinjury is consistent with observed increases in other proteins associated with E‐C coupling after eccentric contraction‐induced muscle injury. We have demonstrated that the nicotinic acetylcholine (Warren et al. 1999) and dihydropyridine (Ingalls et al. 2004) receptors are also upregulated 3–5 days after injury induction. Although we observe enhanced mTORc1 and p70s6k signaling activity 3 days after injury induction (unpublished observation), whether the increases in FKBP12 and nicotinic acetylcholine and DHPRs at this time reflect increased translation or gene expression awaits further study. Nonetheless, the apparent increase in critical E‐C coupling proteins may contribute to the enhanced recovery rate of E‐C coupling failure, while regeneration of damaged sarcomeres appears to take weeks and requires satellite cell involvement (Lowe et al. 1995; Rathbone et al. 2003).

In summary, we document that the content of FKBP12 within anterior crural muscle is reduced immediately after inducing eccentric contraction‐induced muscle injury and that this reduction is significantly correlated with voltage‐gated force production. The observation that FKBP12 content is elevated in the supernatant fraction and restored in the pellet fraction 3 days after injury may suggest that FKBP12 restoration is associated with the relative rapid recovery of E‐C uncoupling compared to the slow restoration of damaged myofibrillar protein. Although we have demonstrated that reductions in junctophilin (Corona et al. 2010) and FKBP12 appear to cause some of the strength deficits associated with eccentric contraction‐induced muscle injury, it is possible that other proteins interacting with the RyR1 may also be disrupted and their loss also contributes to impaired SR Ca^2+^ release and/or force production.

## Conflict of Interest

None declared.
